# Retraction Notice: Suicide Rates by Occupational Group — 17 States, 2012

**DOI:** 10.15585/mmwr.mm6745a6

**Published:** 2018-11-16

On July 1, 2016, *MMWR* published “Suicide Rates by Occupational Group — 17 States, 2012” (*1*). On June 14, 2018, the authors informed *MMWR* about their concerns regarding the validity of some of the findings in the report, and on June 29, 2018, *MMWR* published “*Notice to Readers:* Ongoing Analysis of Suicide Rates Data by Occupational Group from Results Reported in *MMWR*” (*2*). The analysis is complete, and because the corrections change the conclusions, the original report is retracted.

A new *MMWR* report, “Suicide Rates by Major Occupational Group — 17 States, 2012 and 2015,” is published today (*3*). This report corrects inadvertent errors in the retracted report, uses updated methodology, includes authors from the National Institute for Occupational Safety and Health and additional authors from the National Center for Injury Prevention and Control, and provides analysis of both 2012 and 2015 National Violent Death Reporting System data. Corrected errors include the manual misclassification of some occupation codes in the earlier report (e.g., erroneous coding of farmers to the Farming, Fishing, and Forestry major occupational group instead of to the correct Management major occupational group), which led to errors in reporting of suicide numbers and rates in some groups.
